# Smoking-related gestures and anxiety: a preliminary study in treatment-seeking smokers

**DOI:** 10.3389/fpubh.2025.1665612

**Published:** 2025-10-08

**Authors:** Lorenzo Zamboni, Francesca Locatelli, Roberta Vesentini, Rebecca Casari, Alessio Congiu, Anna Guerra, Daniela Bettoni, Silvia Carli, Sara Cappelletti, Silvia Melchiori, Simone Campagnari, Giuseppe Verlato, Maurizio Valentino Infante, Fabio Lugoboni

**Affiliations:** ^1^Department of Internal Medicine, Unit of Addiction Medicine, Azienda Ospedaliera Universitaria Integrata, Verona, Italy; ^2^University of Verona, Verona, Italy; ^3^Diagnostics and Public Health-Unit of Epidemiology and Medical Statistics, University of Verona, Verona, Italy; ^4^Centro Trentino di Solidarietà, Trento, Italy; ^5^Department of Internal Medicine, Azienda Ospedaliera Universitaria Integrata, Verona, Italy; ^6^Thoracic Surgery Department, University and Hospital Trust-Azienda Ospedaliera Universitaria Integrata, Verona, Italy

**Keywords:** tobacco, nicotine, gesture, cigarette, anxiety, addiction, smoking cesation

## Abstract

**Background:**

The act of smoking is not solely driven by nicotine dependence, but also involves behavioral and sensorimotor components that may independently contribute to addiction maintenance. Among these, gestural rituals such as holding and bringing the cigarette to the mouth may play a role in emotional regulation and self-soothing, particularly in individuals with anxiety. This study aimed to explore the relationship between smoking-related gestures, nicotine dependence, and anxiety symptoms in a sample of smokers seeking addiction treatment.

**Methods:**

A total of 81 treatment-seeking smokers were recruited from an Addiction Medicine Unit. Nicotine dependence was assessed using the Fagerström Test for Nicotine Dependence (FTND), anxiety symptoms were measured with the Beck Anxiety Inventory (BAI), and the importance of smoking-related gestures was rated on a 7-point Likert scale via a single-item question. Correlational and non-parametric tests (Spearman’s rho, Kruskal–Wallis, Fisher’s exact test) were used to analyze associations among the variables.

**Results:**

A significant positive correlation was found between anxiety levels and the perceived importance of smoking gestures (*ρ* = 0.254, *p* = 0.0224). No significant association emerged between BAI categorical levels and FTND categories (*p* = 0.346). A trend toward significance was observed between anxiety severity and FTND levels [χ^2^(4) = 8.521, *p* = 0.0742], but no significant correlation was detected between gesturality and FTND measures.

**Conclusion:**

These preliminary findings suggest that smoking-related gestures may be particularly salient for individuals with elevated anxiety, potentially functioning as ritualized coping behaviors independent of nicotine intake. This dimension may help explain why some smokers struggle to quit despite low biochemical dependence. Future studies should develop validated instruments to assess smoking-related motor patterns and explore tailored interventions targeting the behavioral components of tobacco use.

## Introduction

The World Health Organization states that smoking is the leading preventable cause of premature death (1). Globally, cigarette smoking accounts for 10% of all annual deaths and significantly raises the risk of numerous serious diseases (2). In 2012, the global economic burden of smoking, including direct medical expenses and lost productivity, amounted to $1,436 billion, representing 1.8% of the world’s annual GDP (3).

These statistics highlight the crucial role of smoking cessation programs (4, 5) in addressing the widespread consequences of tobacco use. By encouraging individuals to quit smoking, these programs reduce smoking-related healthcare costs and contribute to measurable improvements in population health. In addition to economic considerations, supporting smoking cessation has tangible public health benefits, such as reducing hospital admissions and improving quality of life in the long term. Most importantly, quitting smoking leads to substantial health benefits, including a reduced risk of life-threatening illnesses such as lung cancer, cardiovascular diseases, and respiratory conditions. Therefore, investing in and promoting smoking cessation efforts is essential for achieving long-term public health and economic sustainability.

Tobacco addiction is a complex health issue, and several studies have explored its comorbidities with mental health disorders. Psychiatric disorders—including anxiety, bipolar disorder, insomnia, major depressive disorder, posttraumatic stress disorder (PTSD), suicide attempts, and schizophrenia—are frequently observed among individuals who smoke, suggesting a strong association between tobacco use and mental health conditions (6).

Treatment outcomes among individuals with psychiatric comorbidities tend to be significantly less favorable compared to those observed in the general population. This is particularly evident in the case of depressive disorders, where the presence of depression at the initiation of treatment has been identified as a potential predictor of reduced likelihood of smoking abstinence at a one-year follow-up. Nevertheless, substantial improvements in depressive symptoms have been reported in patients who successfully quit smoking and maintained continuous abstinence for at least 1 year, highlighting the potential bidirectional relationship between smoking cessation and mental health outcomes (7, 8).

In the scientific literature, it is possible to find several studies on the role of tobacco substitutes. These studies aim to examine coping mechanisms for conditioned smoking cues by replicating certain rituals associated with smoking, such as the hand-to-mouth action. Because of this, users now perceive e-cigarettes as a potentially more appealing alternative to smoking compared to low-toxin smokeless tobacco products (9, 10). Several studies have used wearable electronic devices to observe smokers’ gestures (11–14). However, one aspect that is rarely considered and analyzed is the significance of gestural aspects to smokers who want to quit smoking cigarettes.

One aspect that is rarely considered and analyzed is the significance of gestural aspects for smokers who want to quit. For example, the satisfaction derived from using electronic cigarettes can compensate for the need for one’s preferred brand of traditional cigarettes, and the replacement of the ritual associated with smoking gestures and cigarette handling plays a key role (15).

To fully understand this dimension, it is useful to refer to established addiction models, such as the Habit Loop Model. This model describes how a repetitive behavior becomes a habit through a closed-loop cycle (16, 17):

- Cue: A trigger that initiates the behavior. In the case of smoking, this can be a negative emotion like anxiety or stress.
- Routine: The action or behavior that follows the cue. Here, it is the ritualized smoking gesture, such as the hand-to-mouth movement or the manipulation of the cigarette.
- Reward: The benefit that reinforces the routine. The gestures of smoking can provide a calming and soothing effect, independently of nicotine intake, acting as a self-soothing mechanism (15, 18). This behavioral reward can activate dopaminergic circuits in the brain, further reinforcing the habit (16).

Although frequently mentioned in clinical observations and self-reports, smoking-related gestural behavior still lacks a universally accepted conceptual framework. The existing literature offers a fragmented and heterogeneous understanding of this phenomenon. Some studies have focused primarily on the cigarette as an object, emphasizing its symbolic, tactile, and sensory dimensions—often linked to identity, control, and stress regulation (19). In these cases, the cigarette itself may act as a transitional object, offering comfort or a sense of grounding in emotionally charged situations.

Other research has directed attention to the visual and olfactory experience of tobacco smoke itself, describing it as a key component of the gratification process, often associated with relaxation, esthetic appreciation, or even social signaling (20, 21). The sensory presence of smoke may serve to reinforce the habit through immediate feedback and environmental interaction, which can increase its rewarding nature.

A further strand of research has explored the repetitive motor act of bringing the cigarette to the mouth, interpreting it as a ritualistic or self-regulatory gesture. This motor pattern has been compared to behaviors that provide soothing effects through repetition and predictability, such as those observed in certain compulsive or stress-reducing routines (22, 23). Such gestures may fulfill roles beyond nicotine intake, contributing to emotional regulation and embodiment in the smoking experience.

Despite these important contributions, a comprehensive, integrative, and widely accepted definition of tobacco-related gesturality has yet to be established within the scientific community. Further interdisciplinary research is needed to clarify the phenomenological, psychological, and neurobehavioral components of this dimension of smoking, which may play a crucial role in both dependence and cessation processes.

This preliminary study aims to evaluate the relevance of smoking-related gestures in tobacco users before starting a smoking cessation treatment in a Hospital Addiction Unit, observing which characteristics could correlate with the gestures. Our objective is to explore the relationship between gestures, nicotine dependence, and anxiety, providing a perspective that aligns with the Habit Loop Model and its clinical application.

## Materials and methods

The study was conducted from 01/06/24 to 31/12/24.

The Fagerström Test for Nicotine Dependence (FTND) is a six-question questionnaire used to assess the level of nicotine dependence in smokers. The test assigns scores to each answer, and the total score is used to categorize the smoker’s dependence level.

Scoring and Interpretation:

- 0–2 points: Very low dependence.
- 3–4 points: Low dependence.
- 5 points: Moderate dependence.
- 6–7 points: High dependence.
- 8–10 points: Very high dependence.

With regard to the Beck Anxiety Inventory (BAI) (24), only the ordinal variable (total score) was used in the analyses, and it was compared with smoking gesturality and the Fagerström dependence classes. If the categorical form of the BAI variable is to be used, the classification should be defined as follows:

BAI Score Classification

- 0–7: minimal anxiety
- 8–15: mild anxiety
- 16–25: moderate anxiety
- 26–63: severe anxiety

The gestural aspect of smoking behavior was assessed using a single-item question rated on a 7-point Likert scale. The item was: “When you smoke, how important is the gesture for you (e.g., holding the cigarette in your hand, bringing it to your mouth)?”

### Statistical analysis

Categorical variables are reported as absolute values and percentages.

Continuous variables are expressed as mean ± standard deviation or median [interquartile range] if the distribution is not normal.

Associations between categorical variables, classifications of nicotine dependence based on FTND Scores and Fargestrom questionnaire’s question “How soon after you wake up do you smoke your first cigarette?” and gesturality were evaluated using Kruskal-Wallis test.

The relations between gesturality and Beck Anxiety Inventory (BAI) score, Fargestrom questionnaire’s question “How many cigarettes per day do you smoke?,” age and gender were evaluated using Spearman rank correlation coefficient.

A *p*-value < 0.05 was considered statistically significant.

Statistical analysis was performed using Stata 18 (Stata Corporation, College Station, Texas).

## Results

The final sample comprised 81 participants who were evaluated for tobacco use and associated behavioral and psychological variables. As part of the analysis, the relationship between anxiety levels and smoking-related gestural behavior was examined using Spearman’s rank-order correlation. The test yielded a statistically significant positive correlation between anxiety scores, as assessed by the Beck Anxiety Inventory (BAI), and the degree of involvement in gestural components of smoking behavior (Spearman’s *ρ* = 0.254, *p* = 0.0224). This result suggests that individuals with higher levels of anxiety are more likely to engage in pronounced or ritualized smoking-related gestures, such as the hand-to-mouth movement or the manipulation of the cigarette, regardless of nicotine intake.

To further investigate whether anxiety severity was associated with the level of nicotine dependence, participants were categorized into five groups according to their scores on the FTND: Very Low, Low, Medium, High, and Very High dependence. Descriptive analysis of BAI scores across these groups showed a non-linear but overall increasing pattern in anxiety levels corresponding to higher nicotine dependence severity. The mean BAI scores were as follows: Very Low dependence (Mean = 7.00, SD = 4.34, *n* = 6), Low (Mean = 6.58, SD = 7.03, *n* = 19), Medium (Mean = 13.00, SD = 8.39, *n* = 15), High (Mean = 10.97, SD = 9.21, *n* = 32), and Very High dependence (Mean = 10.22, SD = 5.72, *n* = 9). The descriptive analysis of BAI scores across these groups showed a non-linear but overall increasing pattern in anxiety levels corresponding to higher nicotine dependence severity, with mean scores being notably higher in the moderate dependence group (Mean = 13.00, SD = 8.39, *n* = 15) compared to the very low and low dependence groups (Mean = 7.00, SD = 4.34, *n* = 6 and Mean = 6.58, SD = 7.03, *n* = 19, respectively). While a non-parametric Kruskal-Wallis test did not detect statistically significant differences across all five FTND categories [χ^2^(4) = 8.521, *p* = 0.0742], the observed trend suggests a potential relationship that warrants further investigation, particularly with a larger sample size and more targeted statistical analysis.

Additionally, to explore the association from a categorical perspective, both anxiety and nicotine dependence scores were classified into ordinal categories. BAI scores were grouped into four severity classes: Very Low, Medium, High, and Very High. These were cross-tabulated with FTND classes, and Fisher’s exact test was applied to assess the independence of the two variables. The test revealed no statistically significant association between anxiety class and nicotine dependence category (*p* = 0.346), suggesting that the categorical distribution of anxiety levels was not dependent on the degree of tobacco dependence.

In summary, the results of this preliminary analysis highlight a significant relationship between anxiety and the behavioral gesturality associated with smoking, indicating that psychological factors such as anxiety may play a role in maintaining certain motoric components of the smoking ritual. Conversely, no statistically significant association was observed between anxiety and nicotine dependence severity, whether examined through continuous scores or categorical classifications.

## Discussion

This study reveals a statistically significant association between anxiety levels and smoking-related gestural behavior, while no significant relationship was found between anxiety severity and the degree of nicotine dependence, as measured by the FTND. These findings suggest that gestural components of smoking—such as the hand-to-mouth movement, object manipulation, or ritualized motor routines—may reflect a dimension of tobacco use that is functionally distinct from the pharmacological need for nicotine.

While traditional models of tobacco addiction have focused heavily on nicotine dependence and withdrawal (25), the present results highlight the importance of considering gesture as a clinically meaningful behavior that contributes to smoking maintenance. The absence of a strong link between gesturality and nicotine dependence severity implies that gestural behavior may persist independently of biochemical reinforcement, thereby complicating cessation attempts in individuals with high anxiety. Clinically, this may help explain why smokers with strong ritualistic habits often fail to respond to pharmacotherapy alone (26).

The observed correlation between anxiety and gestural involvement is consistent with literature suggesting that some smokers use the act of smoking not solely to deliver nicotine, but as a means of regulating internal emotional states (18). The repetitive, predictable nature of smoking gestures may provide sensory and proprioceptive feedback that reduces physiological arousal, functioning as a form of behavioral self-soothing (15). In some cases, the handling of the cigarette and the physical gestures seem to provide a sense of comfort that goes beyond nicotine delivery.

This study reveals a statistically significant association between anxiety levels and smoking-related gestural behavior, while no significant relationship was found between anxiety severity and the degree of nicotine dependence, as measured by the FTND. These findings suggest that the gestural components of smoking—such as the hand-to-mouth movement, object manipulation, or ritualized motor routines—may reflect a dimension of tobacco use that is functionally distinct from the pharmacological need for nicotine.

While traditional models of tobacco addiction have focused heavily on nicotine dependence and withdrawal (25), the present results highlight the importance of considering gesture as a clinically meaningful behavior that contributes to smoking maintenance. The absence of a strong link between gesturality and nicotine dependence severity implies that gestural behavior may persist independently of biochemical reinforcement, thereby complicating cessation attempts in individuals with high anxiety. This may help explain why smokers with strong ritualistic habits often fail to respond to pharmacotherapy alone (26).

While our statistical analysis did not reveal a significant association between anxiety and FTND scores, the descriptive data suggest a nuanced relationship. The observed non-linear trend, where mean BAI scores were highest in the moderate dependence group, highlights the complexity of this comorbidity. It is possible that the FTND, which primarily measures physical dependence, does not fully capture the psychological and behavioral dimensions of addiction that may be more closely tied to anxiety. This result further supports the need for a multi-faceted approach to assessing tobacco addiction, moving beyond traditional nicotine-centric tools to include psychological and behavioral factors.

The observed correlation between anxiety and gestural involvement is consistent with literature suggesting that some smokers use the act of smoking not solely to deliver nicotine, but as a means of regulating internal emotional states (18). The repetitive, predictable nature of smoking gestures may provide sensory and proprioceptive feedback that reduces physiological arousal, functioning as a form of behavioral self-soothing (15). In some cases, the handling of the cigarette and the physical gestures seem to provide a sense of comfort that goes beyond nicotine delivery.

Our results align perfectly with the Habit Loop Model. Anxiety serves as the cue that triggers the routine of smoking, which are the repetitive and ritualized gestures (16). These gestures, in turn, provide a reward of self-soothing that is sufficient to maintain the habit, even in the absence of high biochemical nicotine dependence. The gestural repetition in smoking may activate dopaminergic loops involving the basal ganglia, contributing to behavioral reinforcement even in the absence of nicotine (16). The link with anxiety suggests further involvement of limbic structures such as the amygdala and anterior cingulate cortex, particularly in individuals with high trait anxiety or affective dysregulation (17).

From a treatment perspective, this differentiation between dependence-related and anxiety-related components of smoking behavior suggests the need for more nuanced cessation approaches. Smokers who present with high anxiety levels and strong gestural habits may benefit from interventions that incorporate anxiety-reduction techniques, such as cognitive behavioral therapy or mindfulness-based stress reduction, alongside strategies aimed at replacing or reframing the motor aspects of smoking (19, 27).

From a treatment perspective, this differentiation between dependence-related and anxiety-related components of smoking behavior suggests the need for more nuanced cessation approaches. Smokers who present with high anxiety levels and strong gestural habits may benefit from interventions that incorporate anxiety-reduction techniques, such as cognitive behavioral therapy or mindfulness-based stress reduction, alongside strategies aimed at replacing or reframing the motor aspects of smoking.

The use of devices such as e-cigarettes or non-nicotine inhalers may provide a transitional solution by mimicking the gestural elements without reinforcing nicotine dependence (19, 27). However, it is crucial to adopt a nuanced view, acknowledging both the short-term utility and potential long-term risks associated with these products. While they may serve as a valuable harm reduction tool, particularly for smokers with high anxiety and strong gestural dependence, they are not a definitive cessation solution. The sustained use of nicotine-containing e-cigarettes, for instance, perpetuates dependence, shifting the behavior rather than eliminating it. Therefore, a comprehensive treatment plan should consider their short-term role in addressing the behavioral component, with a clear strategy for subsequent nicotine reduction and eventual cessation. This balanced approach is essential for supporting a true transition away from nicotine.

Importantly, such approaches should be considered adjunctive rather than primary for patients whose smoking behavior is largely driven by emotional regulation needs.

These findings also support a multidimensional view of tobacco addiction, in line with biopsychosocial and neurobehavioral models that include affective and sensorimotor pathways in the maintenance of drug-seeking behavior (17). Gestural repetition in smoking may activate dopaminergic loops involving the basal ganglia, contributing to behavioral reinforcement even in the absence of nicotine (16). The link with anxiety suggests further involvement of limbic structures such as the amygdala and anterior cingulate cortex, particularly in individuals with high trait anxiety or affective dysregulation.

One of the broader implications of this study is the need for more refined assessment instruments that can capture the role of gesturality in tobacco addiction. Current tools like the FTND or DSM-based criteria focus predominantly on biochemical and withdrawal dimensions, neglecting behavioral rituals that may play a pivotal role in relapse risk. The development of a valid and reliable “Gestural Smoking Scale” could enhance future research and clinical assessment in this area, supporting the development of tailored interventions that decouple the sensorimotor experience of smoking from nicotine delivery.

## Limitations

This study presents several limitations that should be considered when interpreting the findings. First, the cross-sectional design limits the ability to infer causal relationships between anxiety levels and smoking-related gestural behaviors. While a significant association was observed, it is not possible to determine whether increased anxiety leads to greater reliance on gestural rituals, or whether habitual gestural smoking behavior contributes to maintaining higher levels of anxiety. Longitudinal studies are necessary to clarify the directionality of this relationship.

Second, the sample was drawn from individuals accessing services at a hospital-based Addiction Medicine Unit, which may introduce selection bias. In addition, the sample consisted exclusively of individuals seeking treatment at a hospital-based Addiction Medicine Unit, which may not reflect the broader population of smokers. This selection bias could limit the generalizability of the findings to community-based or non-treatment-seeking individuals. Participants in this setting may differ in important ways—such as motivation to quit, psychological comorbidities, or treatment-seeking behavior—from the broader population of tobacco users. As a result, the generalizability of the findings to community samples or less treatment-engaged smokers may be limited.

Third, the gestural component of smoking behavior was assessed through observational and self-report data rather than via standardized or validated instruments. While the study highlights the clinical relevance of gesturality, the lack of a formal, psychometrically tested scale for smoking-related gestures may limit the precision and reproducibility of the findings. The development of a validated “Gestural Smoking Scale” could enhance future research and clinical assessment in this area.

BAI is a widely used and validated instrument for measuring anxiety symptoms, it captures general rather than context-specific anxiety. It is possible that state anxiety specifically related to smoking cues or withdrawal—rather than trait anxiety more broadly—would show an even stronger association with gestural behavior.

Fifth, the study relied on the FTND to assess tobacco dependence severity. While FTND is a widely adopted tool, it primarily reflects physical dependence and does not capture psychological or behavioral components of addiction, including habit strength, emotional triggers, or environmental reinforcement. This may partly explain the lack of correlation observed between FTND scores and gesturality.

Despite these limitations, this study offers a novel perspective that may inform the development of tailored interventions and future research instruments.

## Conclusion

In sum, this study suggests that smoking-related gestures are significantly associated with anxiety, but not with nicotine dependence severity. These findings support the clinical relevance of behavioral rituals in smoking maintenance and cessation, especially among individuals with high anxiety levels. Further studies are needed to better define and quantify the role of gestures in smoking behavior.

## Data Availability

The raw data supporting the conclusions of this article will be made available by the authors, without undue reservation.
